# Functional decline in nonagenarians admitted for hip fracture

**DOI:** 10.1590/1806-9282.20240796

**Published:** 2025-03-17

**Authors:** Sonia Jiménez-Mola, María Plaza-Carmona, Francisco Javier Idoate Gil, Pilar Sáez-López, Paula Fernández García, Jesús Seco-Calvo

**Affiliations:** 1 University Health Care Complex of León – León, Spain.; 2 University Health Care Complex of León, Universidad Internacional de la Rioja – León, Spain.; 3 University Health Care Complex of León, Geriatrics Service – León, Spain.; 4 University Hospital Fundación Alcorcón – Alcorcón, Spain.; 5 University of León, Institute of Biomedicine – León, Spain.

**Keywords:** Hip fractures, Musculoskeletal diseases, Nonagenarians, Frail elderly

## Abstract

**OBJECTIVE:**

The objective of this study was to determine the characteristics of nonagenarian patients admitted for hip fracture and assess whether they present differences in complications and functional outcomes at discharge compared to younger patients.

**METHODS:**

Prospective longitudinal study in patients over 75 years of age admitted for hip fracture over a 1-year period. A total of 542 patients were included, of which 165 patients were aged over 90 years (30.9%). Demographic variables, pre-fracture functional status, medical history, type of fracture, functional evolution, discharge destination, and mortality were collected. Differences between the two population groups were analyzed.

**RESULTS:**

Of the 542 patients over 75 years admitted for hip fracture, 165 were aged over 90 years (30.9%), 74.5% were women, 62% were independently ambulatory prior to the fracture, and 70% had a Barthel Index score >60. Cognitive impairment was absent in 49% of cases. Surgical treatment was performed in 91.5% of cases. There were no differences in the baseline status when compared to younger patients (aged 75–89 years) except for a higher likelihood of residing in a nursing home (OR 1.74; CI 1.18–2.55). Nonagenarian patients were at higher risk of not being able to walk at discharge (OR 2.00; CI 1.29–3.10). Discharge to a nursing home or functional recovery unit was more likely (OR 1.85; CI 1.22–2.81).

**CONCLUSION:**

Patients over 90 years of age are more susceptible to worsening their function during admission and have a higher risk of not being able to walk at discharge. Efforts should be made to reverse this decline in order to reduce the associated mortality.

## INTRODUCTION

In Spain, as in most industrialized countries, life expectancy has increased due to progress in healthcare, social, and economic conditions, and improvements in lifestyle. The life expectancy of the Spanish population at the age of 65 years is among the highest in both the European Union and the world, for both men and women^1^.

Hip fracture is a prevalent condition in older individuals, with 85.4% of all hip fractures occurring in those over 75 years of age^2,3^. It constitutes the most serious clinical complication of osteoporosis, as patients who suffer from it have high morbidity and mortality^4^. It is estimated that the continuous increase will reach 4.5 million hip fractures worldwide by the year 2050^5^. In nonagenarian patients, reduced physiological reserve and the presence of multiple comorbidities and polypharmacy make them more susceptible to complications and a decline in previous function^6^, which may result in the inability to return to their usual residence^7,8^. In this regard, it is crucial to properly initiate the process to treat hip fractures in this age group. Performing surgical intervention within the first 24 or 48 h from hospital admission can decrease the likelihood of patient mortality and improve their functional quality^9^.

Functional decline in the elderly is closely related to increased 1-year mortality following surgical intervention. Some studies on hip fractures in patients over 90 years old, published between 2013 and 2020, have reported a mortality rate ranging from 23.4 to 42.6% per year in this age group^10^.

The average number of complications presented by these patients has been consistently identified as the main associated risk factor for mortality and loss of functionality in the hospital and after discharge^10^. However, it is important to note the lack of research on functional outcomes and mortality related to hip fractures in nonagenarian patients^11^.

The aim of this study was to describe the characteristics of patients over 90 years of age admitted for hip fracture in our hospital and to determine if the age of these patients influences complications and functional outcomes at hospital discharge.

## METHODS

### Study type: inclusion and exclusion criteria

Prospective observational study. A sample of 542 cases (with an average age of 84.2 years) of patients aged from 75 to 105 years consecutively admitted for osteoporotic hip fracture to the Traumatology Service of CAULE from December 2018 to November 2019 was available. Of these, 165 (30.9%) were aged over 90 years. Pathological fractures, periprosthetic fractures, and those resulting from high-intensity traumas were excluded.

### Ethical considerations

The study was conducted in accordance with Spanish legislation and legal regulations governing clinical research on humans (Royal Decree 223/2004, on the regulation of clinical trials), as well as following the Ethical Standards recognized by the 1975 Declaration of Helsinki (last revision carried out in October 2000 at the 52nd General Assembly in Edinburgh). It was approved by the Ethics Committee of the Complejo Asistencial Universitario de León (Ethics-ULE-004-2015). Participants and/or their reference caregiver signed informed consent. All participants were informed of their freedom to withdraw from the study at any time.

### Variables

These were collected from the assessment performed by the specialist geriatrician. Sociodemographic variables, type of fracture, average, and pre-surgical stay were recorded. The patient's previous functional status was assessed using the Barthel Index (0–20, total; 21–60, severe; 60–90, moderate; >90, independent). Walking capacity includes independent/1 cane; 2 canes/walker; substantial assistance; and non-ambulatory. Information on the cognitive status was collected from the cognitive assessment at admission and personal history. Four groups were established: no cognitive impairment, mild cognitive impairment without meeting dementia criteria, moderate dementia, and severe dementia. Place of residence includes own home, with family, and nursing home.

The surgical details recorded included whether surgical intervention was performed, the reason for not proceeding with surgery (if applicable), the anesthetic risk according to the ASA classification, the type of surgical treatment, and the anesthesia used.

At discharge, destination [home, functional recovery unit (FRU), and nursing home], walking ability, and change of address were recorded.

### Statistical analysis

SPSS 27.0 software (IBM SPSS Inc., Chicago, IL, USA) was used for the analysis of the collected variables. The normality of distribution was assessed using the Kolmogorov-Smirnoff test. In the descriptive analysis, quantitative variables are expressed as mean±standard deviation. Categorical variables are presented as percentages or proportions. Means of quantitative variables were compared using the Student's t-test for normally distributed data and the Mann-Whitney U test or Kruskal-Wallis test when they did not adhere to normality. The chi-square test was used to compare proportions.

Binary logistic regression was used to assess different factors associated with being over 90 years of age. First, a univariate study of all factors (taken one by one) was conducted to determine which of them were significantly associated with the category of >90 years. Subsequently, multiple logistic regression was applied to establish a model or profile of risk patients.

## RESULTS

Among the 542 patients aged over 75 years admitted for hip fracture, 165 were aged over 90 years (30.9%). Table 1 describes baseline, surgical, and discharge characteristics. It is noteworthy that 62% were independently ambulatory before the fracture, 70% had a Barthel Index >60, and no cognitive impairment was present in 49%. Surgical treatment was performed in 91.5% of patients (n=151). Among the 14 patients not operated on, 8 died prior to surgery, 3 received orthopedic treatment, and 3 were deemed high surgical risk (non-displaced intracapsular fracture and previous non-ambulation). In-hospital mortality was 9.1% (n=15 patients). At discharge, only 23% were capable of independent ambulation with a walker. However, 37% were transferred to an FRU, 27% returned home, 35% were discharged to a nursing home, and 8% changed their place of residence (Table 1).

**Table 1:** Descriptive analysis.

Variables associated with the baseline situation			
Variable		Number of cases	Incidence (%)
Women/men		123/42	74/24
Barthel			
	Total dependency (≤20)	20	12.1
	Severe dependency (21–60)	29	17.6
	Moderate dependency (61–90)	62	37.6
	Independence (>90)	54	32.6
	Independent ambulation/1 stick	102	61.8
	Walking frame/2 sticks	47	28.5
	A lot of help/does not walk	16	9.7
	Own address	54	32.7
	With family members	57	34.5
	Nursing home	54	32.7
	Not cognitive impairment	81	49.1
	Leve	42	25.5
	Moderate	36	21.8
	Severo	6	3.6
Type of fracture			
	Persubtrochanteric	97	59
	Subcapital	68	41
ASA			
	II	30	18.2
	III	113	68.5
	IV	22	13.3
**Descriptive analysis of variables associated with surgery and discharge**			
**Variable**		**Number of cases**	**Incidence (%)**
Surgery (yes)		151	91
In-hospital mortality		15	9
Surgical treatment			
	Nail	90	59
	Hemiarthroplasty	57	38
	Cannulated screws	4	2.6
Ambulation at discharge			
	Independent ambulation/1 stick	0	0
	Walking frame/2 sticks	34	22.7
	A lot of help	44	29.3
	Does not walk	72	48
Discharge			
	Own address	15	10
	With family members	26	17.3
	Nursing home	53	35.3
	Functional recovery unit	56	37.3

Table 2 summarizes the univariate analysis of baseline factors. No statistically significant differences were found in the pre-fracture functional status or ambulation among patients over 90 years when compared to younger ones. There was no difference in the presence of cognitive impairment. However, two significances were found. The first was not living in one's own home (OR 1.7; CI 1.18–2.55). The second, complementing the former, was living in a nursing home (OR 1.63; CI 1.08–2.44). The rest of the variables studied, such as average and pre-surgical stay, type of anesthesia, and ASA, were not significantly associated (p>0.05). In the study of complications, in univariate analysis (Table 2), significance was found for p<0.01 in renal function impairment, which was 1.97 more likely in those over 90 years (CI 1.25–3.12), as well as in the development of heart failure, which was 2.04 more likely in those over 90 years (CI 1.20–3.46). Significance was also found for p<0.05 in mortality, which was 2.21 more likely in those over 90 years (CI 1.06–4.58).

**Table 2:** Univariate logistic regression.

Relationship of baseline factors with being >90 years old							
Predictor factor	Coefficient B	E.T. (B)	OR	95%CI of OR	Wald	p-value	R^2^
Sex (female)	-0.013	0.215	0.99	0.65–1.51	0.00	0.951NS	0.000
Barthel	-0.075	0.090	0.93	0.78–1.11	0.69	0.407NS	0.001
Deambulation	0.090	0.140	1.09	0.83–1.44	0.41	0.522NS	0.001
Own address (no)	0.552	0.196	1.74	1.18–2.55	7.93	0.005**	0.015
With family members (yes)	0.153	0.199	1.17	0.79–1.72	0.60	0.440NS	0.001
Nursing home (yes)	0.486	0.207	1.63	1.08–2.44	5.51	0.019*	0.010
Cognitive impairment	0.132	0.110	1.14	0.92–1.42	1.43	0.998NS	0.003
**Relationship of complications factors with being >90 years old**							
Anemia	0.355	0.305	1.43	0.79–2.59	1.36	0.244NS	0.003
Transfusion	0.100	0.191	1.11	0.76–1.61	0.28	0.600NS	0.001
Delirium	0.278	0.192	1.32	0.91–1.92	2.08	0.149NS	0.004
Constipation	0.144	0.223	1.16	0.75–1.79	0.42	0.519NS	0.001
Renal function alteration	0.679	0.233	1.97	1.25–3.12	8.46	0.004**	0.015
Urinary tract infection	0.198	0.255	1.22	0.74–2.01	0.60	0.438NS	0.001
Infection/respiratory insufficiency	0.372	0.254	1.45	0.88–2.38	2.16	0.142NS	0.004
Malnutrition	0.082	0.268	1.09	0.64–1.84	0.10	0.758NS	0.000
Heart failure	0.710	0.271	2.04	1.20–3.46	6.88	0.009**	0.012
Acute retention of urine	0.056	0.319	1.06	0.57–1.98	0.03	0.860NS	0.000
Ischemic heart disease	-0.762	0.428	0.47	0.30–0.98	3.17	0.075NS	0.007
Death	0.791	0.372	2.21	1.06–4.58	4.52	0.034*	0.008
Pressure ulcers	0.116	0.473	1.12	0.44–2.84	0.06	0.806NS	0.000
**Relationship of discharge status factors with being >90 years old**							
Destination on discharge: nursing home	0.274	0.208	1.32	0.88–1.98	1.73	0.188NS	0.003
Discharge destination: functional recovery units	0.346	0.206	1.41	0.94–2.12	2.82	0.093NS	0.006
Discharge destination: Nursing home or functional RECOVERY units.	0.613	0.214	1.85	1.22–2.81	8.25	0.004**	0.017
No change of address	-0.230	0.198	0.79	054–1.17	1.36	0.244NS	0.003
Yes change of address	-0.323	0.348	0.72	0.37–1.43	0.86	0.353NS	0.002
Change domic. to functional recovery units	0.378	0.206	1.46	0.97–2.19	3.36	0.067NS	0.007
Ambulation on discharge: a lot of help	0.251	0.219	0.19	0.84–1.97	1.31	0.252NS	0.003
Ambulation on discharge: does not walk	0.381	0.197	1.46	0.99–2.16	3.73	0.053NS	0.008
Ambulation on discharge: a lot of help/does not walk	0.692	0.225	2.00	1.29–3.10	6.47	0.002**	0.020
Weight bearing not permitted	0.324	0.277	0.38	0.80–2.38	1.37	0.241NS	0.003

NS: not significant (p>0.05).

* Significant at 5% (p<0.05).

** Highly significant at 1% (p<0.01).

CI: confidence interval; OR: odds ratio.

In the univariate analysis of variables at discharge (Table 2), significance was found for p<0.01 in discharge destination: Nursing home or FRU, which was 1.85 more likely in those over 90 years (CI 1.22–2.81), as well as in ambulation at discharge: substantial assistance/no ambulation, which was 2.00 more likely in those over 90 years (CI 1.29–3.10).

To conclude this statistical analysis, an attempt was made to find a multiple model that determines the profile of those over 90 years based on the previously related factors. The final model, whose result is summarized in Table 3, contains five of these predictors: ambulation at discharge with substantial assistance or no ambulation: OR 1.97; p=0.006; previous treatment with benzodiazepines: OR 1.75; p=0.012; no previous diagnosis of osteoporosis: OR 0.45; p=0.039; no antidementia treatment: OR 0.36; p=0.013; and no comorbidity with Parkinson's disease: OR 0.15; p=0.013.

**Table 3:** Multiple logistic regression model.

Variables included	Category	Regression model coefficients					
		B	E.T. (B)	Wald	P-sig	OR	95%CI of the OR
Discharge ambulation	A lot of help/does not walk	0.679	0.245	7.68	0.006**	1.97	1.22–3.19
Benzodiazepines	Yes	0.557	0.222	6.29	0.012*	1.75	1.13–2.70
Comorbidity Parkinson's disease	No	-1.922	0.776	6.13	0.013*	0.15	0.03–0.67
Anti-dementia treatment	No	-1.031	0.416	6.13	0.013*	0.36	0.16–0.81
Previous diagnosis of osteoporosis	No	-0.800	0.388	4.25	0.039*	0.45	0.21–0.96
Population constant		-1.430	0.223	41.27	0.000**	–	–
	**Summary of the final model**						
	Omnibus test model significance:				χ^2^=62.23; p=0.000**			
	Test H.L.				χ^2^=1.17; p=0.948NS			
	Adjustment R^2^ Nagelkerke:				0.176			

NS: not significant (p>0.05).

* Significant at 5% (p<0.05).

** Highly significant at 1% (p<0.01). DV: Age over 90 years. CI: confidence interval; OR: odds ratio.

## DISCUSSION

Patients over 90 years, although starting from a similar ambulation status as younger patients, are more likely to experience functional deterioration during hospitalization and have a higher risk of not regaining ambulation upon discharge.

In the study of Leur^12^ on nonagenarian patients, the baseline characteristics of the 165 patients over 90 years admitted for hip fracture in our hospital, predominantly women, with good pre-fracture functional status and independent ambulation, are similar to those reported in studies on patients over 75 years in our setting^13^. There were differences neither in pre-fracture ambulation or functional status nor in the presence of cognitive impairment compared to younger elderly (75–89 years).

However, residing in nursing homes was more common^1^. Despite no differences in pre-surgical stay compared to younger patients, it falls short of recommendations. Delayed surgery is associated with increased infections and worse functional outcomes^14^. Nonagenarian patients have lower physiological reserves, making them more susceptible to complications and pre-existing functional decline^6^.

In our study, patients over 90 years, despite starting from an acceptable ambulation status (62% independent and 28% with technical assistance), are at a higher risk of not regaining ambulation upon discharge (OR 2.00; CI 1.29–3.10). After hip fracture, 78% of patients do not walk or do so with great difficulty at discharge. Several studies have shown that this functional loss is associated with 1-year mortality, with walking capacity after fracture being the most important predictor of 1-year survival^5^.

According to Clinical Practice Guidelines^15^, rehabilitation after hip fracture should begin upon admission, under a multidisciplinary approach. Early mobilization, weight-bearing, and active rehabilitation of the affected limb are the bases of functional recovery, resulting in lower mortality at 6 months and improved ambulation between 2 and 6 months^16^. In our study, although sitting and early weight-bearing were initiated in 88% of patients over 90 years, rehabilitation was not performed during hospitalization. We know that nonagenarian patients benefit from acute and subsequent subacute rehabilitation if they have not achieved their goals^14,17,18^. In the study by Torpilliesi et al. ^19^, a high proportion of nonagenarians were able to achieve independent ambulation after rehabilitation.

In Scarano's study^20^, another difference we found in nonagenarians compared to younger patients in our study, likely related to functional decline, is the higher probability of discharge to a nursing home or FRU (OR 1.85; CI 1.22–2.81). Upon discharge, 37% of nonagenarian patients are transferred to an FRU. The benefits of these units in terms of functional improvement, prevention of nursing home admission, and lower mortality are well established and carry a Jovell^21^ recommendation. However, at times, as indicated by Sáez-López et al.^22^, the lack of availability or absence of these FRUs as a specific Geriatric resource leads to the use of rehabilitation in nursing homes as the only available option, even if it is not recommended by the Geriatric Assessment Team.

Regarding the in-hospital mortality of nonagenarian patients, studies are inconclusive regarding the impact of age^6,8,11^. In our sample, mortality is high (9.1%), with more than half of them dying prior to surgery due to the exacerbation of pre-existing conditions. Age over 90 years was associated with a higher risk of mortality (OR 2.21; 95%CI 1.06–4.58); however, this result was not confirmed in the multivariate analysis.

## CONCLUSION

Patients over 90 years, although starting from a similar ambulation status as younger patients, are more likely to experience functional deterioration during hospitalization and have a higher risk of not regaining ambulation upon discharge. Hence, appropriate resource management should be undertaken to reverse this decline and attempt to reduce the associated mortality.

In this regard, it is essential to emphasize the importance of a comprehensive geriatric assessment upon admission and discharge from the hospital, which allows the design of an individualized preventive, therapeutic, and rehabilitative plan, in order to achieve the highest level of independence and quality of life for the elderly.
